# Erythrophagocytosis in *Entamoeba histolytica* and *Entamoeba dispar*: A Comparative Study

**DOI:** 10.1155/2014/626259

**Published:** 2014-06-05

**Authors:** Daniel Talamás-Lara, Bibiana Chávez-Munguía, Arturo González-Robles, Patricia Talamás-Rohana, Lizbeth Salazar-Villatoro, Ángel Durán-Díaz, Adolfo Martínez-Palomo

**Affiliations:** ^1^Department of Infectomics and Molecular Pathogenesis, Center for Research and Advanced Studies, IPN, Avenida Instituto Politécnico Nacional No. 2508, Colonia San Pedro Zacatenco, Delegación Gustavo A. Madero, 07360 Mexico City, DF, Mexico; ^2^Faculty of Superior Studies Iztacala, Biology, UNAM, Los Reyes Iztacala, 54090 Tlalnepantla, MEX, Mexico

## Abstract

*Entamoeba histolytica* is the causative agent of human intestinal and liver amebiasis. The extraordinary phagocytic activity of *E. histolytica* trophozoites has been accepted as one of the virulence mechanisms responsible for their invasive capacity. The recognition of the noninvasive *Entamoeba dispar* as a different species has raised the question as to whether the lack of pathogenic potential of this ameba correlates with a limited phagocytic capacity. We have therefore compared the process of erythrophagocytosis in both species by means of light and video microscopy, hemoglobin measurement, and the estimation of reactive oxygen species (ROS). In the present study, we confirmed that *E. dispar* has lower erythrophagocytic capacity. We also observed by video microscopy a new event of erythrocyte opsonization-like in both species, being more characteristic in *E. histolytica*. Moreover, *E. dispar* showed a lower capacity to produce ROS compared with the invasive species and also showed a large population of amoebae that did not engulf any erythrocyte over time. Our results demonstrate that *E. histolytica* has a higher phagocytic capacity than *E. dispar*, including a higher rate of production of ROS in the course of ingesting red blood cells.

## 1. Introduction

*Entamoeba histolytica*, an enteric parasite capable of invading intestinal mucosa and spreading to other organs, mainly the liver, is a significant source of morbidity and mortality in developing countries [1, 2]. The motile form of the parasite, the trophozoite, usually lives as a harmless commensal in the lumen of the large intestine where it multiplies and differentiates into a cyst, the resistance form, responsible for transmission of the infection. Occasionally, trophozoites would invade the intestinal mucosa and produce dysentery or* amoeba* and spread to other organs [3]. The existence of two different species of* Entamoeba*, initially proposed by Brumpt in 1925, was approved at the XIII Seminar on Amebiasis, held in Mexico City [4]. At present, unequivocal evidence for the existence of two morphologically similar and closely related species of* Entamoeba* in humans has been substantiated by immunological, genetic and molecular studies. As a result,* E. histolytica*, the invasive organism was formally redescribed to separate it from the noninvasive, and more common,* Entamoeba dispar* [5].

*E. histolytica* invasion starts when trophozoites residing in the colon deplete the mucus, interact with enterocytes, dismantle cell junctions, and lyse host cells [6], whereas* E. dispar* does not break down the mucus barrier or cause epithelial cell damage when in contact with cells on human colonic explants [7].

Although erythrophagocytosis has been proposed as a qualitative pathogenicity indicator rather than a quantitative virulence indicator [8], this process is widely considered as one of the most prominent characteristics of* E. histolytica* virulence [9–11].

Biochemical changes accompanying the process of endocytosis include increases of oxygen and glucose consumption, the activity of the pentose or hexose monophosphate cycle, and hydrogen peroxide production. Together, these changes constitute the respiratory burst [12]. Once phagocytosis occurs, the respiratory burst is carried out as part of the metabolic processes to remove endocytosed material.

Having the opportunity to compare invasive versus noninvasive parasites, we decided to analyze comparatively the erythrophagocytosis process to determine possible differences between these two species of* Entamoeba*. The erythrophagocytic process was registered with light microscopy by means of the Novikoff et al. [13] staining to quantify the amount of ingested erythrocytes and to determine whether the erythrophagocytic capacity of each species correlates with the surface area of each amoeba as determined by light microscopic measurements. We have also used spectrophotometry to analyze the amount of ingested hemoglobin. To corroborate the percentage of nonphagocytic populations, video microscopy, flow cytometry, and confocal microscopy were used. Moreover, the extent of the respiratory burst, using NBT, was measured for each amoebic species.

## 2. Materials and Methods

### 2.1. Cells

Amoebas were cultured in borosilicate glass tubes under axenic conditions.* E. histolytica* trophozoites HM1-IMSS species were grown to logarithmic phase (72 h) in TYI-S-33 medium at 36°C [14] and* E. dispar* trophozoites in YI-S culture medium [15] for 72 h at 36°C. Both culture media contained 10% bovine serum and a vitamin mixture. Parasites were harvested by chilling the culture tubes at 4°C in a water-ice bath for 10 min and then they were centrifuged at 900 ×g for 5 min. Type B human erythrocytes (Rh+) were freshly obtained in Alsever's solution (Sigma Aldrich Company, UK) and washed 3 times in the same solution to remove white blood cells. The erythrocytes were counted and used in a 1 : 100 (trophozoites : erythrocytes) ratio in quantitative erythrophagocytosis assays and 1 : 5 ratio for video microscopy analysis of erythrophagocytosis. Yeasts of the genus* Candida albicans* species CAI4 generated from SC5314 (Clinical Systemic Isolate) [16] were routinely maintained in YPD medium [17]. Minimal defined medium consisted of 2% glucose supplemented with yeast nitrogen base (DIFCO). After that,* Candida albicans* were washed 2 times and harvested in phosphate buffered saline solution (PBS) and centrifuged at 900 ×g for 5 min and finally counted and used at a 1 : 100 (trophozoite : yeast) ratio in phagocytosis assays to measure the respiratory burst.

### 2.2. Erythrophagocytosis

*E. histolytica* and* E. dispar* trophozoites were washed in TYI-S-33 and YI-S without bovine serum, respectively. To establish the interaction, erythrocytes were added, and the interaction was carried out for 5, 10, and 15 min at 37°C without bovine serum, using a 1 : 100 amoeba-erythrocyte ratio for quantitative studies and 1 : 5 amoeba-erythrocyte ratio for video microscopy studies. Analysis was done with the AxioVision SE64 software with images obtained with a Zeiss Axiophot photomicroscope.

### 2.3. Quantitative Erythrophagocytosis, Study, and Correlation of Ingestion/Area

For quantitative experiments, 900 *μ*L (5 × 10^5^ amoebas) was incubated with 100 *μ*L (5 × 10^7^ erythrocytes) at different times, at 37°C. At the end of the incubation time, amoebas were resuspended in 1 mL of distilled water to lyse free erythrocytes and stop erythrophagocytosis. Trophozoites of both species can resist the osmotic shock without alterations of the plasma membrane permeability. Cells were centrifuged at 900 g/min for 5 min and pellets were fixed with 2.5% glutaraldehyde in PBS. Phagocytosed erythrocytes were visualized by phase contrast microscopy with the alkaline benzidine method as described [13]. Amoebas were incubated for 30 min at 37°C in 2 mL of 3,3-diaminobenzidine (Sigma) at a concentration of 2 mg/mL, 0.2% H_2_O_2_ in 0.05 M 2-amino-2-methyl-propanediol-HC1 (Merck-Schuchardt) at pH of 9.7. After washing with PBS, the number of erythrocytes present in the cytoplasm of 100 amoebas, in each time, was counted in triplicate. Considering that* Entamoeba* strains do not contain peroxisomes [18], cytoplasmic components positive to benzidine were considered only as ingested erythrocytes. Correlation tests regarding ingestion/area were conducted by measuring the surface area of each amoeba and the number of erythrocytes phagocytosed, using the AxioVision software SE64.

### 2.4. Indirect Determination of Erythrophagocytosis (Quantification of Hemoglobin)

For a precise analysis of erythrophagocytosis, a quantitative determination of hemoglobin was done. Nonfixed trophozoites were washed with Turk's solution to eliminate noningested erythrocytes; trophozoites were pelleted and lysed with 1 mL of formic acid and the amount of hemoglobin was measured by spectrophotometric analysis at 400 nm.

### 2.5. Video Microscopy

Amoebas (1.25 × 10^5^) were placed on coverslips resuspended in 100 *μ*L of the respective medium and then erythrocytes (6.25 × 10^5^) were added in 5 *μ*L of Alsever's solution, maintaining slide temperature at 37°C. Micrographs and video micrographs sequences were taken with a Zeiss Axiophot microscope.

### 2.6. Fluorescent Labeling of Cells

To avoid manipulation of erythrocytes, 15 *μ*L of whole blood were resuspended in Alsever's solution (300 *μ*L) and labeled with 2.5 *μ*L of Sytox 9 through incubation for 1 h at room temperature under constant stirring. After that, cells were washed twice at 600 ×g for 10 min and finally erythrocytes were resuspended in 1.5 mL Alsever's solution.

### 2.7. Flow Cytometry and Confocal Microscopy

The existence of a nonphagocytic population in both species was quantitative and qualitatively determined by flow cytometry and confocal microscopy respectively. By flow cytometry the number of amoebas that had ingested stained erythrocytes was evaluated at different times, as mentioned. Once the time had elapsed, cells were centrifuged at 900 ×g for 10 min and then fixed with freshly prepared 4% (v/v) paraformaldehyde, for 1 h. After that, cell suspensions were washed 4 times with PBS and then read in a flow cytometer FACS-Calibur (Becton Dickinson). Confocal microscopy was used to distinguish ingested erythrocytes from free erythrocytes and to show amoebas that had not phagocytosed. Coverslips were mounted with Vectashield (Vector Laboratories; Ontario, Canada) and analyzed by confocal microscopy in an LSM700 microscope (Carl Zeiss Microimagin GmbH, Carl Zeiss, Germany).

### 2.8. Reduction of Nitroblue Tetrazolium (NBT) to Assess the Phagocytic Function

Amoebas (3.5 × 10^5^/400 *μ*L) previously adhered for 15 min to coverslips were incubated with yeast (3.5 × 10^7^/100 *μ*L) for 30, 60, and 120 min by the addition of 500 *μ*L of NBT (1 mg/mL in sterile PBS) [19]. At the end of interaction, the reaction was stopped by adding 1 mL of 70% methanol dissolved in PBS for 10 min and washing once with PBS to remove excess of methanol; immediately after, 0.5% safranin dissolved in water was added and incubated for 1 min. The excess was removed with several washes until the sample was slightly stained. Coverslips were mounted, and yeast that had been reduced was counted using a phase contrast microscope. Counts were done taking 100 amoebas randomly, in triplicate, for each processed sample, and color changes that had occurred inside of cells were counted, taking as a positive value at least 1 single reduced yeast, and amoebas that did not show a color change in their cytoplasm were taken as a negative value. This method was performed in triplicate with only 2 possible variants (positive and negative) whereby only positive and negative cells where counted in each time.

### 2.9. Statistical Analysis

In the data obtained concerning the number of phagocytosed erythrocytes, absorbance of hemoglobin, and amoebas that reduced NBT when exposed to pathogenic yeast at different times with each species of* Entamoeba*, the following descriptive measures were calculated: average, minimum, and maximum standard deviation, quartiles (Q1, Q2, Q3), and coefficient of variation (Data not shown). Likewise, box and whisker diagrams were developed to further describe the phenomena occurring in the process of erythrophagocytosis and phagocytosis. To determine whether significant differences were present in the number of ingested erythrocytes, absorbance of hemoglobin, and amoeba showing NBT reduction, factorial analysis of variance was applied (ANOVA) with the transformed data of these variables (data were transformed using the Box-Cox technique because the Anderson-Darling test indicated that data were not normal). Once the ANOVA test was performed to establish which conditions showed differences, the Tukey's test was applied to measure the difference in the mean values between groups. These statistical analyses were carried out using MiniTab software version 16.0.

## 3. Results 

A comparative analysis of the phagocytic capacity of* E. histolytica* and* E. dispar* may help to understand the mechanisms involved in the virulence of* E. histolytica*. To date limited information exists about the erythrophagocytic capacity of* E. dispar. *Here we demonstrate basic differences in the phagocytic process between these two parasites.

### 3.1. Comparative Analysis of the Erythrophagocytic Capacity between* E. dispar* and* E. histolytica*

#### 3.1.1. Determination of the Number of Ingested Erythrocytes

**Figure 1:**
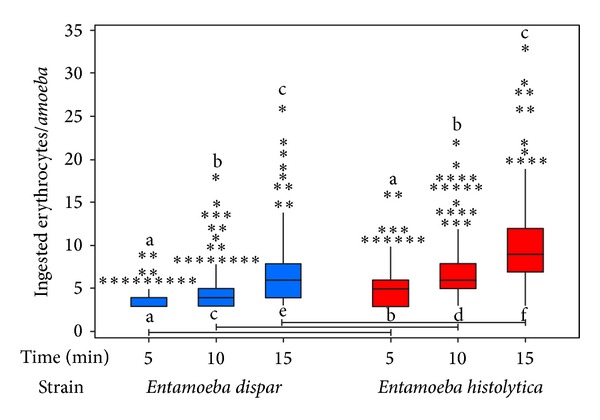
Erythrocyte uptake performed with* E. dispar* (blue) and* E. histolytica* (red) by light microscopy. The experiment was repeated three times independently in triplicates. The statistical comparisons showed significant differences in the erythrophagocytic capacity between species and among times (*P* < 0.001; different letters on top). Moreover, species compared with their reciprocal times also showed a significant difference (*P* < 0.001; bottom letters: a with b, c with d, and e with f) (*). Outliers.

As a first step in the analysis of erythrophagocytosis, the number of ingested erythrocytes per amoeba was determined after 5, 10, and 15 min of interaction. As shown in Figure 1, this is a time-dependent process for both* E. dispar* and* E. histolytica*, with a clear increase in the number of ingested erythrocytes with longer times of interaction. ANOVA test between species and time variables was carried out showing that these variables, “species” and “time,” showed a significant difference (*P* < 0.001). With Tukey's test, each variable with respect to all other variables of “species” and “time” was compared. Results show that, for each reciprocal combination, the *P* value was less than 0.001 (*P* < 0.001). This confirms significant differences among all combinations (Figure 1).

#### 3.1.2. Determination of Hemoglobin Content in Trophozoites

To corroborate the results obtained by direct assays, indirect measurements were carried out by spectrophotometry to determine the absorbance produced by the hemoglobin contained in erythrocytes ingested by 5 × 10^5^ trophozoites of each species at previously described times. Therefore, it was possible to obtain additional and more accurate information about the erythrophagocytosis process. In addition, ANOVA between different variables (species and time) was carried out to determine if differences found in absorbance between* E. histolytica* and* E. dispar *were significant.

**Figure 2:**
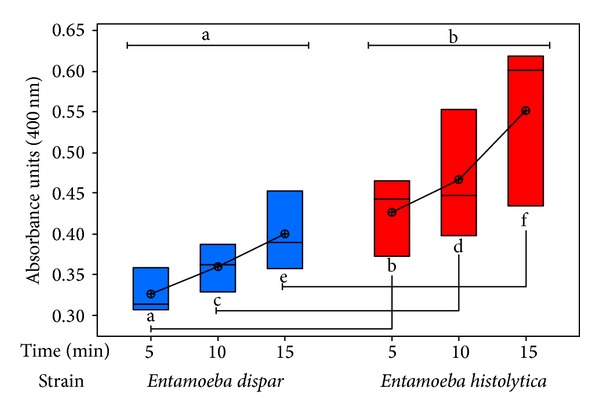
Hemoglobin content in* E. dispar* (blue) and* E. histolytica* (red) after erythrophagocytosis, determined by spectrophotometry. The experiment was repeated three times independently in triplicates. Quantification of erythrophagocytosis measured by ingested hemoglobin indicated no significant differences among times (letters on top, connected by the same line segment; a and b). A significant difference between species compared with their reciprocal times (*P* < 0.001; bottom letters connected by the same line segment) was observed.

As expected, there were clear differences in the erythrophagocytic capacity of* E. dispar* versus* E. histolytica* (*P* < 0.001); however, in contrast to results found when counting erythrocytes, hemoglobin determination did not show significant differences among time in the same species. With Tukey's test, each variable with respect to all other “species” and “time” variables were compared. Results show that, for each reciprocal combination, the *P* value was less than 0.001 (*P* < 0.001) (Figure 2).

#### 3.1.3. Correlation between Trophozoites' Surface Area and Ingested Erythrocytes as a Function of Time

**Figure 3:**
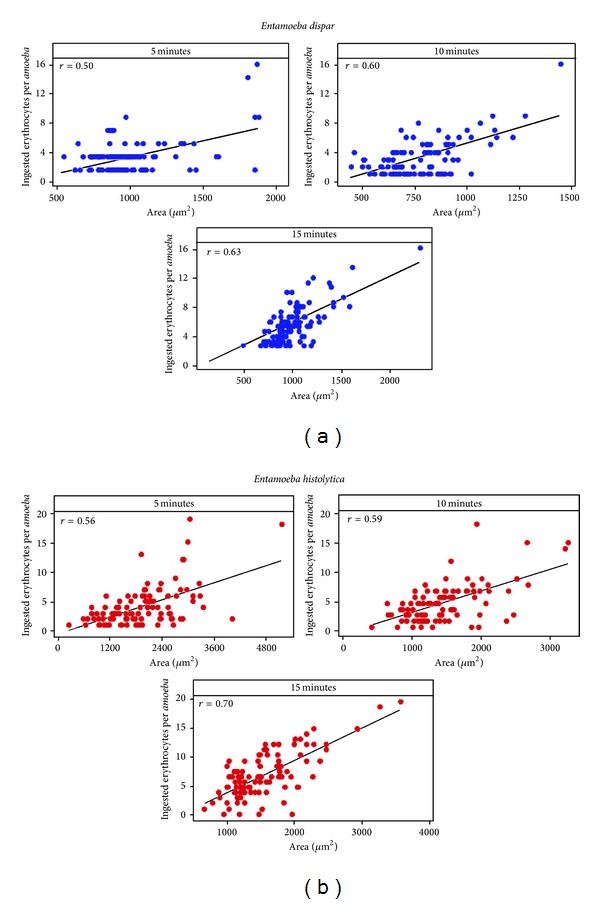
Scatter diagrams showing correlations between area and the number of ingested erythrocytes with respect to interaction time. There is a direct and significant relation (*P* < 0.001) for both strains, meaning that the larger is the area of the amoeba, these engulf more erythrocytes. This ratio increases as time passes. The same trend for both strains was observed; however, correlations were higher for* E. histolytica *(“*r*” value).

To analyze whether the amoeba area could be a factor related with a higher rate of erythrophagocytosis, a dispersion analysis was performed. Results showed a relative, time-dependent association between amoeba area and number of phagocytosed erythrocytes, with “*r*” values of 0.5, 0.60, and 0.63 for* E. dispar* at 5, 10, and 15 min respectively, and with “*r*” values of 0.56, 0.59, and 0.70 for* E. histolytica* at 5, 10, and 15 min, respectively. Therefore, there was a direct relationship between the average surface area of the amoeba and the number of ingested red cells, with a significant difference (*P* < 0.001) between the two species; furthermore, this ratio increased with time. Although the same trend is observed for both species, the correlation values of* E. histolytica* were higher (Figure 3).

### 3.2. Comparative Analysis of the Erythrophagocytic Process between* E. dispar* and* E. histolytica*

#### 3.2.1. Opsonization-Like Event during Erythrophagocytosis

**Figure 4:**
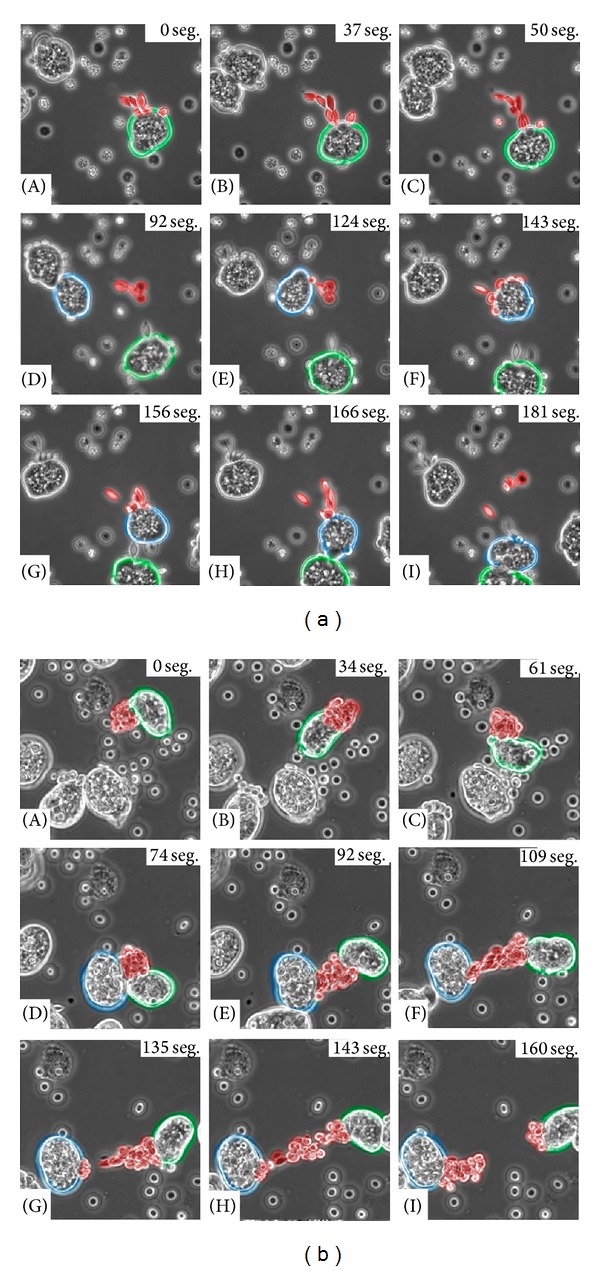
Real time video microscopy showing the opsonization-like event. (a)* E. dispar*: Figure 4(a)(A) shows a trophozoite (green) with a group of bound erythrocytes (red); then it can be seen how these erythrocytes completely detach from this amoeba (Figure 4(a)(B–D)), probably due to a low affinity binding. A new trophozoite (blue) appears in the scene (Figure 4(a)(D)) ready to bind the erythrocytes that had been bound to another amoeba (Figure 4(a)(E and F)). Once again, these erythrocytes will detach from this blue amoeba (Figure 4(a)(G–I)). (b)* E. histolytica*: Figure 4(b)(A) shows a trophozoite (green) which binds erythrocytes (red) sending them to the caudal pole until it encounters another trophozoite (blue) (Figure 4(b)(B–D)); after both amoebas bound to the same group of erythrocytes, it is appreciated how they “fight” to keep the erythrocytes clump (Figure 4(b)(E–H)). Finally, Figure 4(b)(I) shows how every amoeba keeps a portion of the erythrocytes. Numbers shown in the upper right corner of each image correspond to time in seconds.

Erythrophagocytosis by* E. histolytica* has been a widely studied mechanism [8, 9, 11, 20–22]. However, with the aid of video microscopy, a new characteristic of this process was observed. Before describing this event, it is necessary to mention that the adhesion process between* E. dispar* and erythrocytes is rather weak. Apparently, amoebas have an opsonization—like mechanism which consisted in the fact that erythrocytes that had had a previous contact with amoebas were clearly more susceptible to be bound and/or be ingested by other trophozoites. This event was observed with both species, though with clear differences between them.  Figure 4(a)(A) shows how an* E. dispar* trophozoite (green) has adhered to a group of erythrocytes (red) on the caudal pole; as mentioned before, probably due to the weakness of this binding, this group of erythrocytes is released from the amoeba (Figure 4(a)(B–D)); however, these erythrocytes were attracted to another amoeba (blue) (Figure 4(a)(E)), despite having more erythrocytes surrounding it. This amoeba (blue) seems to show some tropism for those erythrocytes that had been previously adhered to the green amoeba; subsequently, the amoeba (blue) adheres to the same group of erythrocytes and continues its mobility. However, this group of red blood cells becomes totally detached from the trophozoite (Figure 4(a)(F–I)).

*E. histolytica* also presented this phenomenon, although the attachment strength was higher than that observed with* E. dispar* due to the apparent morphological distortion and stress (tension) generated in erythrocytes.  Figure 4(b)(A) shows an amoeba (green) with a large number of adhered erythrocytes in the caudal pole that can be tracked all the way until it encounters another amoeba (blue) (Figure 4(b)(B–D)); this second amoeba (Figure 4(b)(D)) had the ability to impressively adhere to the same group of erythrocytes that are being carried by the first amoeba and to generate a competition force to acquire the group of red cells (Figure 4(b)(E–H)); finally a break of this agglomerate occurs, and both amoebas capture a portion of the initial erythrocyte group (Figure 4(b)(I)).

#### 3.2.2. Nonphagocytic Population of Amoebas

**Figure 5:**
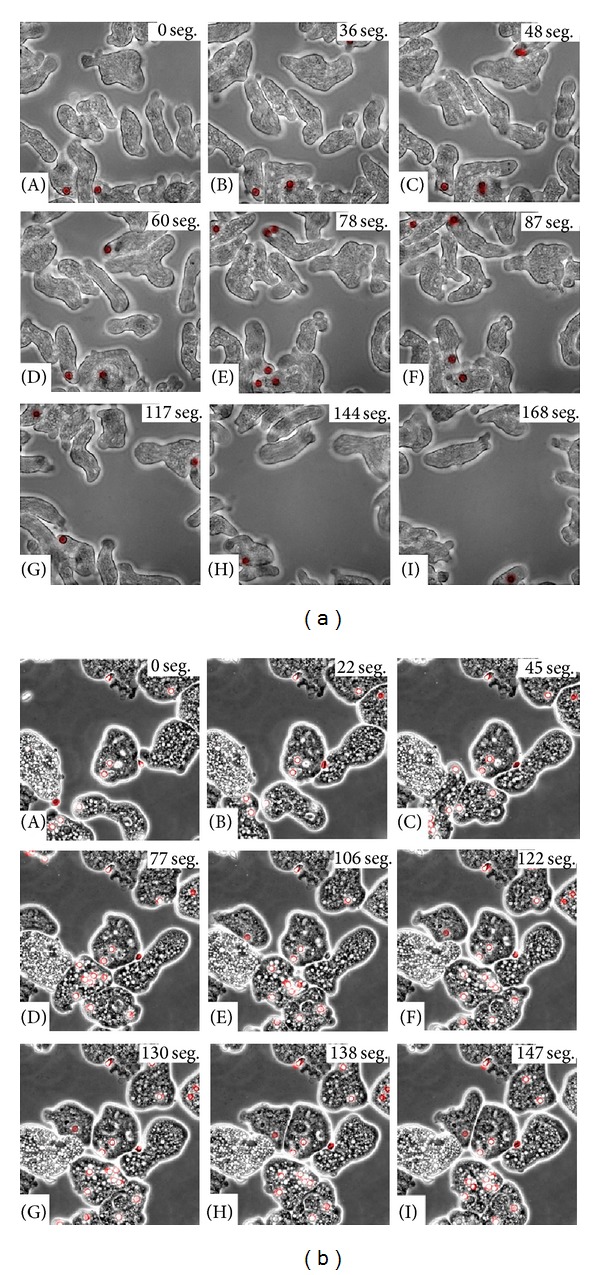
Major nonphagocytic subpopulation of* E. dispar* versus a major phagocytic subpopulation of* E. histolytica*. Images taken from a video microscopy followup of the erythrocytes-trophozoites interaction showing the existence of a nonphagocytic subpopulation of* E. dispar* (most of the cells are nonphagocytic). Only a few cells contain erythrocytes (red dots) inside their cytoplasm even after 20 min interaction ((a)(A–I)). On the contrary, images taken from the video microscopy followup of* E. histolytica* trophozoites show that nearly 90% or higher of the population have ingested erythrocytes (red dots), after only 5 min of interaction ((b)(A–I)). Numbers shown in the upper right corner of each image correspond to time in seconds starting after 20 min incubation for* E. dispar *and 5 min incubation for* E. histolytica*.

Having evidence by video microscopy about the low adhesion capacity of* E. dispar* and observing that there were a notable number of nonphagocytic cells, we decided to determine the percentage of the nonphagocytic population for each species. Results showed that, after 20 to 30 min interaction of* E. dispar* with erythrocytes, (Figure 5(a)(A–I)) only a small number of amoebas with a limited number of internalized erythrocytes (red dots) were observed. In contrast,* E. histolytica* trophozoites, with only 5 min of interaction with erythrocytes (Figure 5(b)(A–I)) showed not only a large number of red blood cells in their cytoplasm (red dots) but also a very small number of amoebas that did not have ingested erythrocytes.

**Figure 6:**
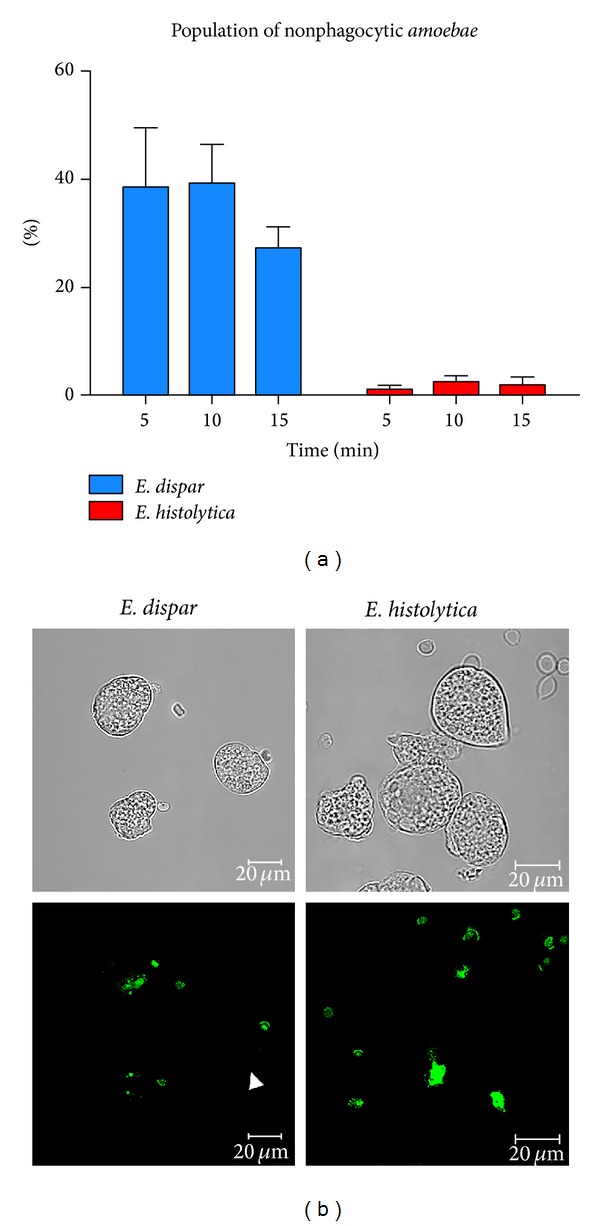
Quantitation and confocal analysis of the nonphagocytic and phagocytic subpopulations of* E. dispar* and* E. histolytica*. Graph showing the percentage of nonphagocytic amoebas after different times of interaction for* E. dispar* (blue) and* E. histolytica* (red) (a). Experiments were done in triplicate for each of the analyzed times. (b) shows representative images of phagocytic and nonphagocytic subpopulations for* E. dispar* (left side) and* E. histolytica* (right side). Upper panels, light microscopy; lower panels, confocal images. Arrow head: a nonphagocytic trophozoite.

Having demonstrated by video microscopy that a nonphagocytic population exists, flow cytometry assays were performed to quantitate this phenomenon. As expected for control assays, only 0.08% from 20,000 events of nonstained erythrocytes, showed some autofluorescence. In contrast, Sytox-stained erythrocytes showed that 99.87% of the cells were positive for Sytox fluorescence; thus Sytox-positive erythrocytes were then used for interaction experiments (data not shown).  Figure 6(a) shows the summary of these experiments where there was a significant difference (*P* < 0.001) between species; however, there were no differences in the times measured. This confirms that nonphagocytic population remains constant regardless of the time. It is important to mention that a viability test, with Trypan blue, was performed on trophozoites populations; they always had a 97% or higher viability (data not shown).

The previous results obtained by flow cytometry were confirmed by confocal microscopy.  Figure 6(b) shows the two species, at 15 min of interaction, and illustrates how the number of ingested erythrocytes by* E. dispar* is smaller than that in* E. histolytica*; also it shows an* E. dispar* trophozoite without any ingested erythrocytes (Figure 6(b), left side, arrowhead); as expected in the* E. histolytica* field, all trophozoites have ingested fluorescent erythrocytes (Figure 6(b), right side).

### 3.3. Comparative Analysis of ROS Production during Erythrophagocytosis by* E. dispar* and* E. histolytica*

**Figure 7:**
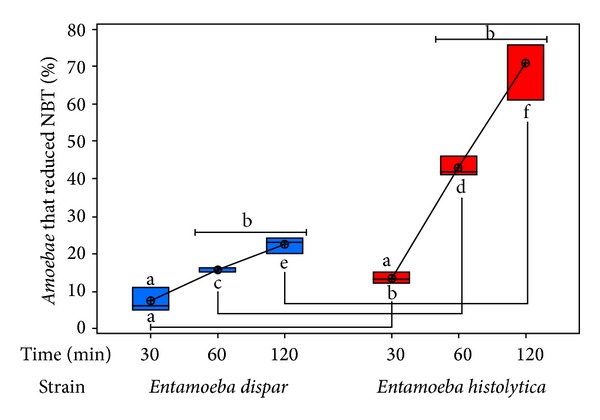
Evaluation of the difference in the oxide-reduction ability between* E. dispar *and* E. histolytica* using the NBT reduction assay. The experiment was repeated three times independently in triplicates. Results describing the oxide-reduction ability of both strains, measured by reduction of NBT, indicate that after 60 min incubation, there is not a significant change in the amount of formazan produced (letters on top connected by the same line segment); however, between 30 and 60 min there is a significant difference appreciated in both species (letters on top). Moreover, species compared with their reciprocal times also showed a significant difference (*P* < 0.001) (different letters on bottom connected by the same line segment).

The redox capability of each species by the NBT technique was analyzed by light microscopy. The presence or the absence of blue formazan in yeast* Candida albicans* inside amoebas was determined in 100 randomly chosen amoebas. Results regarding the variable “species” on the ability to produce reactive oxygen species and the variable “time” required to produce them showed a significant difference (*P* < 0.001). With Tukey's test, each variable with respect to all other variables “species” and “time” was compared. Results show that, for each reciprocal combination, the *P* value was less than 0.001 (*P* < 0.001); however, at 1 and 2 h no significant differences between times for each of the species were found because the maximum value of the respiratory burst occurs during the first hour [23] (Figure 7). Even though there was no significant difference, if we consider the slope, we can see that the number of amoebas that reduce NBT increase with the time much more rapidly in the case of* E. histolytica* in comparison with* E. dispar*.

## 4. Discussion

Phagocytosis is a central feature in the pathogenesis of invasive amebiasis, still an important public health problem [24]. Considering that not all* Entamoeba* species have the same degree of virulence, understanding of the various pathogenic mechanisms is an overriding goal. The unequivocal evidence for the existence of* E. dispar*, an amoeba closely related to* E. histolytica*, but not invasive, opens the way to study differences and similarities shared by the two species of amoebas that colonize the human intestine [25–28].

In this work, we have addressed the study of erythrophagocytosis from three different approaches, quantitative, qualitative, and biochemical, allowing presenting a comprehensive panorama of this phenomenon. We therefore were able to demonstrate the existence of a nonphagocytic subpopulation in each species, much larger, however, in* E. dispar* (40% in* E. dispar* versus 5% in* E. histolytica*). Moreover, a new event, similar to the two species, was the opsonization-like process of erythrocytes; this event was also stronger in* E. histolytica* than in* E. dispar*.* E. histolytica* has a larger capacity to induce phosphatidylserine exposure in the erythrocytes surface while* E. dispar* exhibits a deficient adhesion process with red blood cells and also a very poor induction of phosphatidylserine exposure, resulting in a less efficient phagocytosis [20]. The importance of phosphatidylserine exposure for* E. histolytica* engulfment of host cells has been suggested by the work of Bailey et al., [22], who previously demonstrated that liposomes containing phosphatidylserine or synthetic negatively charged phospholipids, dicetyl phosphate, stimulate* E. histolytica* actin polymerization, a necessary event for efficient phagocytosis.

### 4.1. Erythrophagocytosis

This process is highly asynchronous with many variations that generate high standard deviations; furthermore, when values are analyzed in this way, it is not easy to find statistically significant differences. For this reason, statistical analysis of this study was performed using box and whisker plots; this allowed us to collect information about individual amoebas and not to treat them as a joint population. This gave us the opportunity to identify particular events that usually are not detectable or distinguishable by other techniques, such as the presence of* E. histolytica* amoebas that engulfed 20 or even uncountable erythrocytes; this could be due to the presence of amoebas that ingested erythrocytes more rapidly or to the presence of a subpopulation of larger size as shown by the graphs of scattering values of area versus number of phagocytosed erythrocytes. In the case of* E. dispar*, the average population area ranges from 500 to 1200 *μ*m^2^, and very few amoebas fall out of range while the average for* E. histolytica* population falls in the range of 1000 to 2000 *μ*m^2^ with many more individual amoebas being bigger than 3000 *μ*m^2^.

The spectrophotometric analysis of hemoglobin content, a more sensitive technique used as a complementary approach for erythrophagocytosis evaluation, was useful because samples were treated as total populations and individual variations were overcomed. This might explain why in the graph showing results of hemoglobin content, significant differences among times of interaction were not found within species.

Going back to results obtained by Trissl et al. [9] and comparing their results with ours in terms of the number of ingested cells by the parasites obtained from an asymptomatic carrier, with those obtained in this work, it is clear that their values were similar to those obtained by us with* E. dispar*.

On the subject of the data obtained by flow cytometry, confocal microscopy, and video microscopy, we were able to record a population of nonphagocytic amoebas for each species and their behavior over time, supporting the results obtained by Sateriale et al. [29] who were able to separate amebic subpopulations of* E. histolytica* with higher and lower rates of phagocytosis. When microarrays tests were applied, these authors found that highly phagocytic amoeba showed overexpression of at least 121 genes with respect to nonphagocytic amoeba; therefore, additional studies with* E. dispar* will be necessary to differentiate sub- and overexpressed genes in this species. This confirms the heterogeneity of the amoebic populations that might show different virulence even within the same population. Nowadays, the availability of the genomic sequences of* E. histolytica* and* E. dispar* would allow the analysis of the genetic divergence and differential gene expression between these two species and those genes and molecules associated with the erythrophagocytosis process (adherence, movement, endocytosis, etc.), to identify virulence mechanisms present in the virulent species and absent or not expressed in the nonvirulent parasites [30].

### 4.2. Respiratory Burst

In mammals, the production of reactive oxygen species during phagocytosis has been widely studied and has been associated with the killing capacity of immune cells to destroy microorganisms [31]. In* E. histolytica* there are only a few reports [32, 33] that describe a NBT reduction activity that implies that oxidoreduction activities are essential virulence components. It is known that* E. histolytica* is a highly phagocytic parasite and with respect to* E. dispar* we have shown that these amoebas can also phagocytose erythrocytes, however at a lower proportion; consequently we decided to analyze if the phagocytic process in both* Entamoeba* species would trigger an oxidative burst, when feeding amoebas with pathogenic yeasts* Candida albicans*. Results showed that as low as 30% of* E. dispar* trophozoites were able to reduce NBT, in comparison with* E. histolytica* where as much as 80% of the cells reduced NBT. The conversion of NBT to formazan by the oxidative metabolism of* E. histolytica* and* E. dispar* was used as a viability indicator, to measure their survival after challenge with various antiamoebic drugs [34, 35]. However, to date there are no studies that associate the production of oxygen reactive species during phagocytosis by* Entamoeba* species. On the contrary, the ability of trophozoites to neutralize reactive oxygen species has been characterized and proposed as a virulence mechanism or as a mechanism that provides the trophozoite with a major ability to survive to immune cells attack [36]. Therefore, the results here presented are consistent with the fact that* E. dispar*, being considered a noninvasive amoeba, displays a smaller range of ROS production with respect to* E. histolytica*.

Recent studies have suggested that the generation of ROS and the presence of NADPH oxidase are necessary in cancer cells for the formation of specialized structures called invadosomes (mechanosensory adhesive modules that consist of a dense core filamentous actin surrounded by a ring of adhesion molecules able to infiltrate tissue under physiological and pathological conditions) that allow for a more efficient tissue invasion process [37–40]. In this regard, the possible existence of invadosome-like structures in* E. histolytica* and its lack in* E. dispar* is worth pursuing.

## 5. Conclusions

We performed a comparative study of erythrophagocytosis between* E. dispar*, a noninvasive amoeba, and* E. histolytica*, a highly virulent and invasive parasite. Results demonstrate that the phenomenon is present in both* Entamoeba* species and that there are significant differences between the two amoebas. Both, phagocytosis and the ability to produce reactive oxygen species, are clearly more pronounced in* E. histolytica* in comparison to* E. dispar*.
